# Highly Self-Healable Polymeric Coating Materials with Enhanced Mechanical Properties Based on the Charge Transfer Complex

**DOI:** 10.3390/polym14235181

**Published:** 2022-11-28

**Authors:** Chanjae Ahn, Pyong Hwa Hong, Juhen Lee, Jinsil Kim, Gyeongmin Moon, Sungkoo Lee, In Park, Haksoo Han, Sung Woo Hong

**Affiliations:** 1 Organic Materials LAB, Samsung Advanced Institute of Technology, 129 Samsung-ro, Yeongtong-gu, Suwon-si 16677, Republic of Korea; 2 Department of Chemical and Biomolecular Engineering, Yonsei University, 50 Yonsei-ro, Seodaemun-gu, Seoul 03722, Republic of Korea; 3 Intelligent Sustainable Materials R&D Group, Korea Institute of Industrial Technology, 89 Yangdaegiro-gil, Ipjang-myeon, Seobuk-gu, Cheonan-si 31056, Republic of Korea; 4 Department of Chemical Engineering, University of Montreal, 2900 Edouard Montpetit Blvd, Montreal, QC H3T 1J4, Canada; 5 Current Address: MS Development Team, PI Advanced Materials, 27 Godeung 1-gil, Iwol-myeon, Jincheon-gun, Chungcheongbuk-do 27818, Republic of Korea; 6 Convergence Research Center for Solutions to Electromagnetic Interference in Future-Mobility, Korea Institute of Science and Technology, Seoul 02792, Republic of Korea

**Keywords:** polymer, coating material, self-healing, engineering plastic, charge transfer complex, trade-off

## Abstract

Polymeric coating materials (PCMs) are promising candidates for developing next-generation flexible displays. However, PCMs are frequently subjected to external stimuli, making them highly susceptible to repeated damage. Therefore, in this study, a highly self-healing PCM based on a charge transfer complex (CTC) was developed, and its thermal, self-healing, and mechanical properties were examined. The self-healing material demonstrated improved thermal stability, fast self-healing kinetics (1 min), and a high self-healing efficiency (98.1%) via CTC-induced multiple interactions between the polymeric chains. In addition, it eliminated the trade-off between the mechanical strength and self-healing capability that is experienced by typical self-healing materials. The developed PCM achieved excellent self-healing and superior bulk (in-plane) and surface (out-of-plane) mechanical strengths compared to those of conventional engineering plastics such as polyether ether ketone (PEEK), polysulfone (PSU), and polyethersulfone (PES). These remarkable properties are attributed to the unique intermolecular structure resulting from strong CTC interactions. A mechanism for the improved self-healing and mechanical properties was also proposed by comparing the CTC-based self-healing PCMs with a non-CTC-based PCM.

## 1. Introduction

Polymers can be extensively used in next-generation electronics and transportation applications because they are generally lighter than metals and ceramics, easy to process for mass production, easily combinable with other materials for enhanced performance, and highly versatile [1]. For example, polymeric coating materials (PCMs) are promising candidates for protecting the surfaces of flexible displays (foldable, rollable, wearable, and stretchable displays). However, polymers coated on such products are frequently subjected to mechanical, chemical, thermal, and ultraviolet (UV) stimuli during use. Therefore, to reduce maintenance costs, maintain the original qualities, and extend the lifespan of the final product, self-healing technology, which is capable of autonomous damage repair, must be applied to PCMs [2].

Self-healing technology has been studied using extrinsic and intrinsic approaches [3,4,5]. Extrinsic self-healing materials can repair damage by utilizing microcapsules or microfibers containing a healing agent. Damage causes the capsule or fiber to rupture, releasing healing liquid to repair the damage [6,7,8,9]. Intrinsic self-healing materials exploit reversible interactions between the components in the matrix for self-repair. Reversible interactions are classified as chemical (Diels–Alder reactions [10,11,12] and disulfide bonds [13,14,15]) or physical (host–guest interactions [16,17], hydrogen bonding interactions [18,19,20], ionic interactions [21,22,23], metal–ligand interactions [24,25,26], and π–π interactions [27,28,29]).

While extrinsic self-healing materials lose their ability to mend themselves once the healing agent is depleted during restoration, intrinsic self-healing materials can repeatedly cure damage, even in locations that previously sustained damage and underwent healing. Therefore, PCMs based on intrinsic self-healing systems are more suitable than those based on extrinsic self-healing systems for protecting the surfaces of flexible displays. However, previously reported intrinsic self-healing materials generally exhibited low mechanical strength, suggesting that functional polymers with excellent mechanical strength must be used to develop PCMs that can be practically used for the applications mentioned above.

Engineering plastics (EPs) are high-performance polymers that outperform traditional plastics in terms of their mechanical properties and thermal, chemical, and environmental stabilities [30,31,32]. Typical examples of EPs include polycarbonates, polyamides, polyesters, polyacetals, modified poly(phenylene oxide)s, polyethersulfones, poly(phenylene sulfide)s, polyetherketones, and polyimides. Polyimide (PI), in particular, is known to possess a unique internal structure (a charge transfer complex (CTC)) [33,34,35]. The imide groups in PI have an electron-withdrawing characteristic, owing to which, an electron acceptor, which has relatively fewer electrons, forms between two imide groups. Moreover, an electron donor, which carries an abundance of electrons, is created outside the imide groups. This electron donor–acceptor formation replicates in an alternating pattern across the PI chain. Consequently, a CTC forms via multiple interactions between electron donors and electron acceptors between the PI chains [36,37] (see Figure S1 in Supporting Information for a schematic of the CTC formation). Owing to CTC interactions, PI generally exhibits exceptionally high mechanical strength and thermal stability compared to other EPs.

It has been reported that physical interactions (host–guest, hydrogen bonding, ionic, metal–ligand, and π–π interactions) between the polymeric chains in an intrinsic self-healing system are the main driving force for triggering self-healing [3,4,5,38]. Because CTC interactions are stronger than the typical physical interactions mentioned above, CTC-based self-healing PCMs are expected to possess excellent self-healing ability. In addition, because the enhanced polymeric interchain interactions generally increase the bulk (in-plane) and surface (out-of-plane) mechanical strengths of the polymers [19,39,40], CTC-based self-healing systems can overcome the trade-off between the mechanical strength and self-healing capability in conventional self-healing systems. Therefore, the presence of CTCs is ideal for preparing high-performance self-healing PCMs with outstanding self-healing and mechanical properties. However, to the best of our knowledge, self-healing PCMs solely based on CTC interactions have not yet been reported.

In this study, we present a new class of self-healing PCMs based on CTC interactions to achieve high self-healing efficiency and rapid self-healing kinetics, as well as to eliminate the trade-off exhibited by conventional self-healing materials. Their mechanical properties and self-healing ability were compared with those of conventional EPs in terms of CTC interaction to demonstrate that CTC interactions can be used to develop new high-performance polymeric materials. A mechanism for enhancing the CTC-induced self-healing and mechanical properties was also proposed by controlling the degree of CTC interactions.

## 2. Materials and Methods

### 2.1. Materials

We used 1,2,4,5-Cyclohexanetetracarboxylic acid dianhydride (HPMDA) (Changzhou Sunlight Pharmaceutical Co., Ltd., Changzhou, China, ≥99.24%) 4,4′-(hexafluoroisopropylidene)diphthalic anhydride (6FDA) (Changzhou Sunlight Pharmaceutical Co., Ltd., Changzhou, China, ≥99.74%), 4,4′-oxydianiline (ODA) (Changzhou Sunlight Pharmaceutical Co., Ltd., Changzhou, China, ≥99.86%), 2,2′-ethylenedioxy-bis-ethylamine (EDBEA) (Chemsky International, Shanghai, China, ≥99.0%), and *N,N′*-dimethylformamide (DMF) (Alfa Aesar, Haverhill, MA, USA, ≥99.8%) as received. Polyether ether ketone (PEEK), polysulfone (PSU), and polyethersulfone (PES) were purchased from CS Hyde (Lake Villa, IL, USA).

### 2.2. Preparation of CTC-Based Self-Healing PCMs

Table 1 lists the experimental details and chemical compositions of the CTC-based self-healing PCMs (FE100, FE75, FE50, and FE25). Briefly, 6FDA, EDBEA, ODA, and DMF were added to a double-jacketed reaction flask purged with nitrogen gas. FE100: 6FDA (6.0335 g, 13.58 mmol), EDBEA (2.0129 g, 13.72 mmol), and DMF (76.7 mL); FE75: 6FDA (5.9840 g, 13.47 mmol), EDBEA (1.4973 g, 10.20 mmol), ODA (0.6811 g, 3.40 mmol), and DMF (77.8 mL); FE50: 6FDA (6.0230 g, 13.56 mmol), EDBEA (1.0047 g, 6.85 mmol), ODA (1.3711 g, 6.85 mmol), and DMF (80.1 mL); FE25: 6FDA (5.4721 g, 12.32 mmol), EDBEA (0.4564 g, 3.11 mmol), ODA (1.8686 g, 9.33 mmol), and DMF (74.3 mL). The solid content of each solution was maintained at 20 wt%. Each solution was mechanically stirred using an anchor impeller at 25 °C for 24 h for the condensation reaction. The samples in this study were prepared using a flow coating process. Each solution was homogeneously mixed and degassed using a paste mixer (PDM-300, Dae-Wha Tech., Yongin-si, Republic of Korea) and then coated onto a glass substrate using a film applicator. The coated solution was thermally treated to prepare FE100, FE75, FE50, and FE25 in three consecutive steps (100 °C for 60 min on a hot plate; 25 °C to 200 °C at a heating rate of 5 °C/min in a convection oven; 200 °C for 30 min in a convection oven).

### 2.3. Preparation of Non-CTC-Based PCM

Table 1 lists the experimental details and chemical compositions of the non-CTC-based PCM (HE100). HPMDA (5.0156 g, 22.37 mmol), EDBEA (3.3153 g, 22.59 mmol), and DMF (79.4 mL) were added into a double-jacketed reaction flask purged with nitrogen gas. The solid content was maintained at 20 wt%. The solution was mechanically stirred using an anchor impeller at 25 °C for 24 h and homogeneously mixed and degassed using a paste mixer (PDM-300, Dae-Wha Tech., Yongin-si, Republic of Korea). The resulting solution was coated onto a glass substrate and thermally treated to prepare HE100 in three consecutive steps (100 °C for 60 min on a hot plate; 25 °C to 200 °C at a heating rate of 5 °C/min in a convection oven; and 200 °C for 30 min in a convection oven).

### 2.4. Characterizations

The structural characterization of the samples was performed using a ^1^H nuclear magnetic resonance (NMR) spectrometer (VNMRS 600 MHz, Varian, Crawley, UK) with CD_3_SOCD_3_ (for FE100, FE75, and FE50) and CDCl_3_ (for FE25 and HE100) as the NMR solvents. The thermal properties were measured using thermal gravimetric analysis (TGA, Pyris 1 TGA, Perkin Elmer, Waltham, MA, USA) at a heating rate of 10 °C/min under a nitrogen atmosphere and differential scanning calorimetry (DSC, DSC 8500, Perkin Elmer, Waltham, MA, USA) at a heating rate of 10 °C/min under a nitrogen atmosphere. The self-healing properties were measured using an optical microscope (HT004, Himax-tech, Seoul, Republic of Korea) and by producing scratches using a hardness test pencil (Model 318S, ERICHSEN, Hermer, Germany) with an ISO standard tip (1 mm) and a loading force of 5 N. The self-healing temperatures for FE100, FE75, FE50, FE25, PEEK, PSU, and PES were set to the glass transition temperature *T*_g_ + 20 °C, while that for HE100 was set to *T*_g_ + 40 °C. An alpha stepper (Alpha-Step IQ, KLA-Tencor Corporation, Milpitas, CA, USA) was used to measure the scratch depth. The self-healing efficiency was calculated as follows:

(1) $$\mathrm{Healing}\;\mathrm{Efficiency}\;\left(\%,\;\mathrm{HE}\right)=\frac{S-S_{H}}{S}\times 100$$

where *S* and *S*_H_ are the depths of the scratches before and after self-healing, respectively. The mechanical bulk properties were measured using a universal testing machine (UTM, QM100SE, QMESYS, Norwood, MA, USA) at a rate of 10 mm/min and using a load cell of 50 kN. The specimens were cut into rectangular shapes that were 6 mm wide and 45 mm long. The surface mechanical properties were measured using a nanoindentation tester (NST^3^, Anton Paar, Graz, Austria) with a conical tip of radius 20 μm, a linear loading rate of 6 mN/min from 0 to 1.0 μN, and a scratching velocity of 2 μm/min. Each replicated specimen for measuring the mechanical properties was obtained from a single sample.

## 3. Results and Discussion

### 3.1. Synthesis

**Scheme 1:**
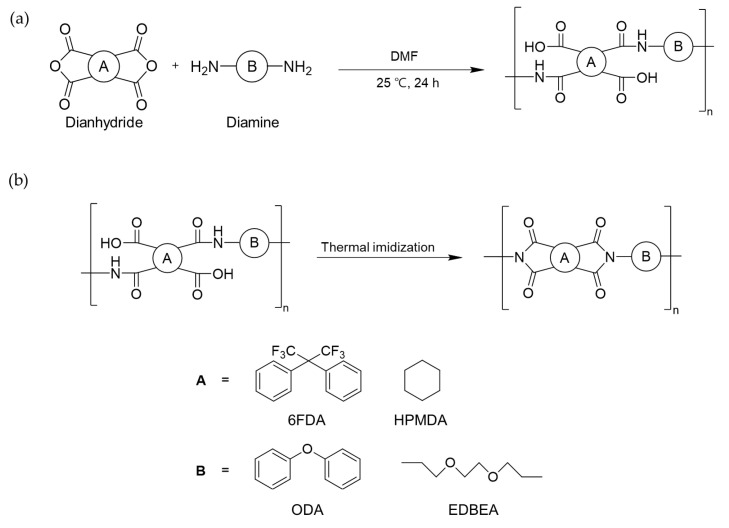
Overall schemes for (**a**) the synthesis of the precursor (poly(amic acid)) of polyimide and (**b**) the thermal conversion of poly(amic acid) to polyimide (FE100, FE75, FE50, FE25, and HE100).

**Figure 1:**
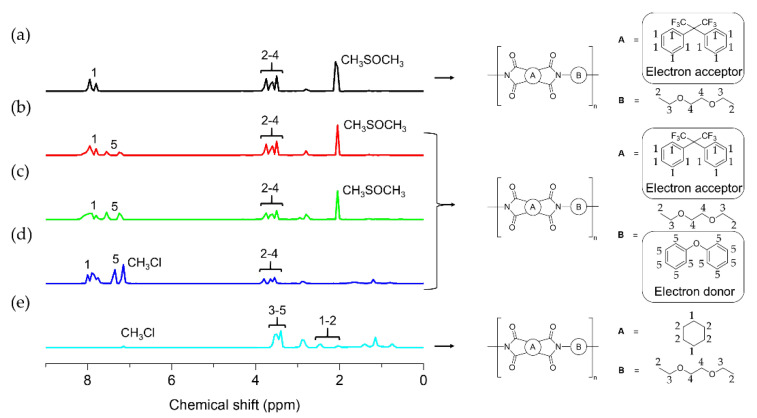
^1^H NMR spectra and chemical structures of (**a**) FE100, (**b**) FE75, (**c**) FE50, (**d**) FE25, and (**e**) HE100.

Scheme 1 illustrates the synthetic routes for the samples, and Table 1 lists the compositions of the samples in this study. The FEX (X = 100, 75, 50, and 25) samples were prepared as follows: 6FDA (100 mol%) with an aromatic structure was reacted with ODA (100–X mol%) with an aromatic structure and EDBEA (X mol%) with an aliphatic structure to form a precursor with amic acid groups. The samples were finally obtained by the thermal imidization of the precursors, in which the amic acid groups were converted into the imide groups via a condensation reaction [34,37]. The structures of the samples were confirmed using ^1^H NMR spectrometry and are shown in Figure 1. While the FE100 spectrum only showed the peaks associated with the protons of aromatic rings in 6FDA (7.7–8.1 ppm), the FE75, FE50, and FE25 spectra showed the peaks associated with the protons of aromatic rings in 6FDA (7.7–8.1 ppm) and ODA (7.0–7.3 ppm). Scheme 1b shows that an electron acceptor (aromatic ring) and an electron donor (aromatic ring) are formed inside and outside the two imide groups, respectively. Consequently, CTC interactions occur through multiple interactions between electron donors and electron acceptors in the polymeric chains. As a reference, HE100 was also prepared using HPMDA (100 mol%) with an alicyclic structure and EDBEA (100 mol%) according to the same method mentioned above. The HE100 spectrum showed only peaks corresponding to the protons of the alicyclic rings in HPMDA (2.1–2.5 ppm), while no proton peaks were observed above 7.0 ppm. In addition, as shown in Scheme 1b and Figure 1e, HE100 has no aromatic rings, indicating that CTC interactions do not occur in HE100. Consequently, FE100, FE75, FE50, and FE25 represent CTC-based self-healing PCMs, whereas HE100 represents a non-CTC-based PCM.

### 3.2. Thermal Properties

**Figure 2:**
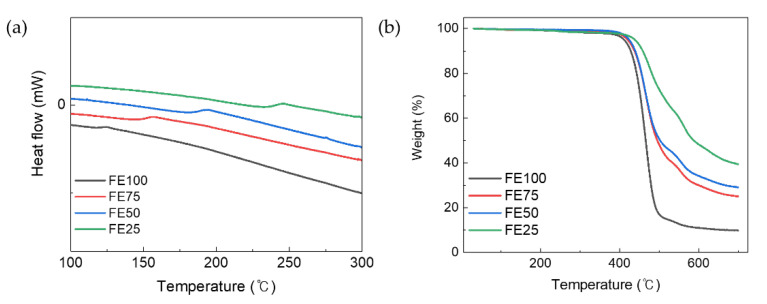
(**a**) DSC thermograms and (**b**) TGA curves of FE100, FE75, FE50, and FE25.

The thermal properties of the samples were measured using DSC and TGA. Figure 2 shows the DSC thermograms and TGA curves, and Table 1 lists the thermal decomposition temperature (*T*_d_) and glass transition temperature (*T*_g_). As shown in Figure 2a and Table 1, the *T*_g_ values increase with increasing amounts of ODA in the order FE100 (120.1 °C) < FE75 (151.3 °C) < FE50 (187.6 °C) < FE25 (240.0 °C). The *T*_g_ values depend on the chemical structure of the monomers: ODA has a rigid aromatic structure, whereas EDBEA has a flexible aliphatic structure. Consequently, as the amount of rigid ODA increases, CTC interactions become stronger and the polymeric chains become stiffer, which causes *T*_g_ to increase. Because HE100 only comprises alicyclic (non-aromatic) and flexible aliphatic monomers, its *T*_g_ (92.6 °C) is significantly lower than those of other samples (see Figure S2 in Supporting Information for the DSC thermogram of HE100).

Figure 2b shows the TGA curves of FE100, FE75, FE50, and FE25. None of the samples show significant weight loss or thermal decomposition when the temperature is under 350 °C. The *T*_d_ values of FE100, FE75, FE50, and FE25 at 2 wt% weight loss are in the ranges of 340–360 °C, 380–400 °C, 390–410 °C, and 390–410 °C, respectively. In addition, the residual weight retention of FE25 at 600 °C is approximately 400% higher than that of FE100. The samples containing higher amounts of ODA exhibit better thermal stability, as shown in Figure 2b. This is because ODA (an aromatic structure) is known to be more planar than EDBEA (an aliphatic structure). Consequently, the sample containing more ODA can form a dense structure because of effective π–π stacking of the planar polymeric chains, which results in significantly enhanced thermal stability.

### 3.3. Self-Healing Properties

**Scheme 2:**
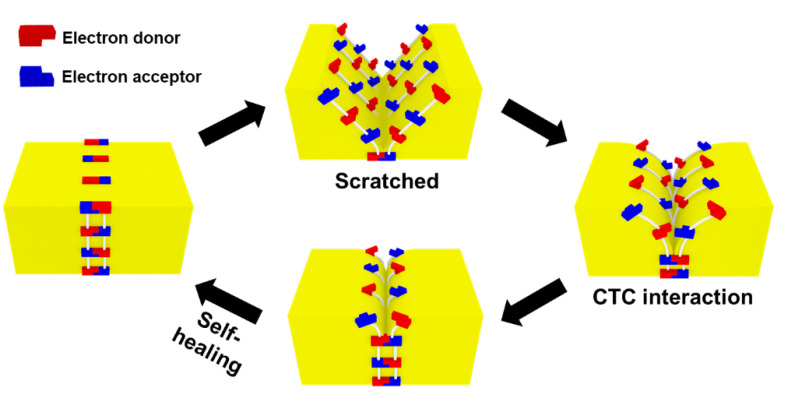
Schematic illustration of the proposed self-healing mechanism.

**Figure 3:**
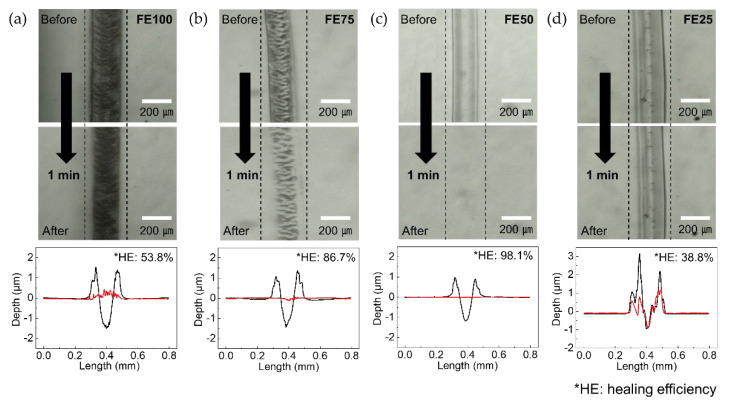
Optical microscope images and depth profiles of (**a**) FE100, (**b**) FE75, (**c**) FE50, and (**d**) FE25 before (0 min; black) and after (1 min; red) the self-healing process. Each self-healing temperature is set to *T*_g_ + 20 °C. The scale bar is 200 μm. HEs were calculated by averaging three measurements after excluding the highest and lowest values.

**Figure 4:**
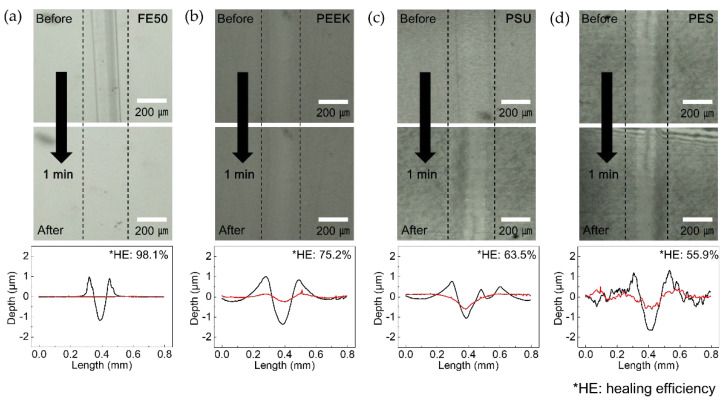
Optical microscope images and depth profiles of (**a**) FE50, (**b**) PEEK, (**c**) PSU, and (**d**) PES before (0 min; black) and after (1 min; red) the self-healing process. Each self-healing temperature is set to be *T*_g_ + 20 °C. The scale bar is 200 μm. HEs were calculated by averaging three measurements after excluding the highest and lowest values.

Figure 3 and Figure 4 show the optical microscope images, depth profiles, and healing efficiencies of FE100, FE75, FE50, FE25, and conventional EPs (PEEK, PSU, and PES) before and after self-healing. The depths of the scratches before and after self-healing were measured by an alpha stepper and were used to calculate the self-healing efficiencies. Aromatic rings are directly responsible for the formation of CTCs [33]; therefore, it is expected that the higher the aromatic ODA content of the sample, the stronger the CTC interactions. Consequently, FE50 (98.1%) has a higher healing efficiency than FE100 (53.8%) and FE75 (86.7%), as shown in Figure 3, because FE50 has more CTC interactions that serve as the main driving force for self-healing. The excellent healing ability of FE75 is attributed to the increased number of interactions between the electron donors and acceptors along the cut surfaces, leading to reformed CTC interactions, as shown in Scheme 2. This will be further discussed in the Proposed Mechanism section. In addition, while the self-healing of FE100 with weak CTC interactions is slow, that of FE50 with enhanced CTC interactions becomes so strong that the damaged section achieves an excellent recovery with 98.1% healing efficiency in 1 min.

Interestingly, FE25 (38.8%) exhibits poor healing efficiency (38.8%) compared with FE50, although FE25 contains more ODA than FE50. As stated previously, the *T*_g_ of FE25 is much higher than that of the other samples, and the self-healing temperature for FE25 (*T*_g_ + 20 °C = 260 °C) is sufficient to trigger the thermal degradation of the polymer during self-healing. Consequently, the decreased CTC interactions caused by the thermally degraded FE25 reduce the self-healing performance of FE25 compared with that of FE50.

The self-healing properties of FE50 were compared with those of commercially available EPs to highlight one of the advantages of self-healing PCMs. As shown in Figure 4, traces of scratches remain on the surface of PEEK, PSU, and PES after self-healing, and the healing efficiencies of PEEK (75.2%), PSU (63.5%), and PES (55.9%) are significantly lower than those of FE50 (98.1%). The *T*_g_ of FE50 (187.6 °C) is similar to those of PSU (190.0 °C) and PES (180.0 °C) and is slightly higher than that of PEEK (151.3 °C), as listed in Table 2. Assuming that the chain mobility of each sample at its self-healing temperature (*T*_g_ + 20 °C) is comparable, it is concluded that, unlike conventional EPs, FE50 demonstrates exceptional self-healing properties because of its CTC-induced specific interactions, emphasizing the importance of CTC for self-healing.

### 3.4. Mechanical Properties

**Figure 5:**
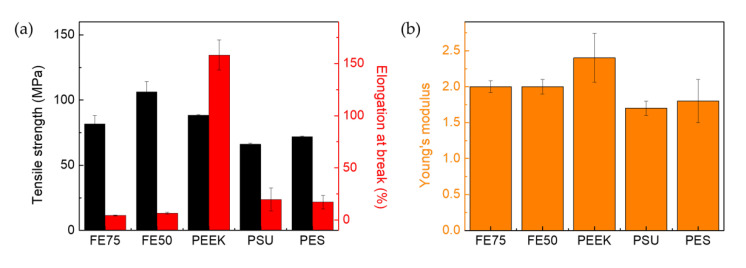
(**a**) Tensile strength (black) and elongation at break (red) and (**b**) Young’s modulus of FE75, FE50, PEEK, PSU, and PES. All data points were obtained by averaging three measurements after excluding the highest and lowest values.

**Figure 6:**
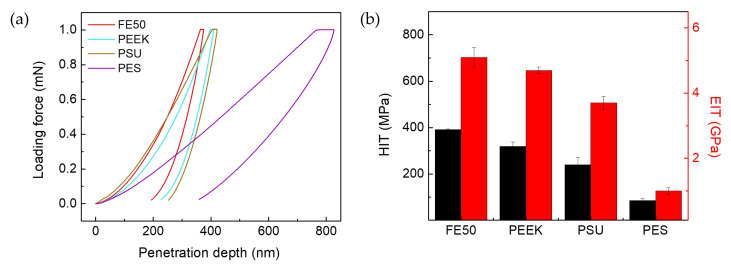
Nanoindentation data of FE50, PEEK, PES, and PSU: (**a**) loading–unloading curves and (**b**) nanoindentation hardness (HIT) (black) and nanoindentation modulus (EIT) (red). All data points were obtained by averaging four measurements after excluding the highest and lowest values.

It has been reported that self-healing materials usually have a trade-off relationship between self-healing ability and mechanical strength [19,20,23]. Figure 5 and Figure 6 illustrate the essential benefits of the self-healing PCMs developed in this study. The bulk (in-plane) and surface (out-of-plane) mechanical properties of FE75, FE50, PEEK, PSU, and PES were measured using a universal tensile machine and nanoindentation tester. All results were obtained by averaging 3–4 measurements after excluding the highest and lowest values.

The detailed bulk and surface mechanical properties are shown in Figure 5 and Figure 6 and Table 2. FE50 displays a higher tensile strength and Young’s modulus than FE75. The increase in the tensile strength and Young’s modulus in FE50 is due to the increased number of CTC interactions between the polymeric chains of FE50. As the ODA concentration increases, the number of electron donors also increases, which is responsible for the increased number of CTC interactions between the electron donors and acceptors. In addition, as shown in Figure 5, FE50 exhibits the highest tensile strength (106.2 MPa) and second highest Young’s modulus (2.0 GPa) compared with conventional EPs. Furthermore, compared with conventional EPs, FE50 has the highest nanoindentation hardness (HIT, 386.0 MPa) and nanoindentation modulus (EIT, 5.3 GPa), as shown in Figure 6b. It has been acknowledged that the mechanical strength of polymers can be significantly increased by tightly packing the polymeric chains through specific interchain interactions [19,38,39]. In contrast with conventional EPs, FE50 exhibits strong CTC-induced interactions that can tightly pack its polymeric chains, leading to improved bulk and surface mechanical properties. The results for the mechanical and self-healing properties of the self-healable PCMs in this study imply that the trade-off exhibited by conventional self-healing materials can be effectively overcome by utilizing CTC interactions.

### 3.5. Proposed Mechanism

**Figure 7:**
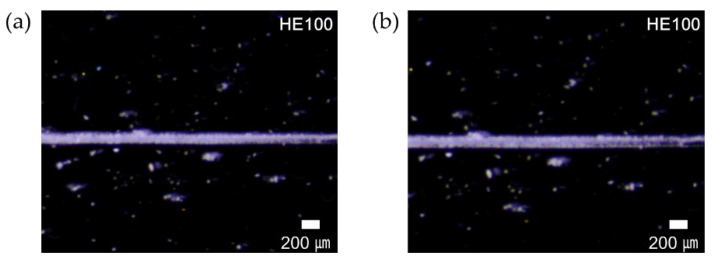
Optical microscope images of HE100 (**a**) before (0 h) and (**b**) after (12 h) the self-healing process. The self-healing temperature is set to *T*_g_ + 40 °C. The scale bar is 200 μm.

A mechanism for the improved self-healing and mechanical properties of CTC-based self-healing PCMs is proposed in this study. For this purpose, a reference PI (HE100) without aromatic rings was synthesized from alicyclic and aliphatic monomers. The absence of aromatic rings in the polymeric chain of HE100 meant that no CTC interactions between the electron donors and acceptors were anticipated between the polymeric chains. HE100 was subsequently employed to prepare a non-CTC-based PCM, and its self-healing properties are shown in Figure 7.

To study the origin of self-healing, the self-healing behaviors of the CTC-based self-healing PCMs and non-CTC-based PCM were compared. The self-healing process of polymeric materials can only take place above the *T*_g_ of the polymers because the polymeric chains require sufficient mobility to make contact with each other to initiate self-healing [41]. Note that the self-healing temperatures of HE100 and FE50 were *T*_g_ + 40 °C and *T*_g_ + 20 °C, respectively, indicating that the thermal mobility of HE100 was much higher than that of FE50. While FE50 demonstrates remarkable self-healing kinetics and recovery from damage (98.1% healing efficiency within 1 min), as shown in Figure 3 and Figure 4, HE100 shows no evidence of self-healing, as shown in Figure 7 (see Figure S3 in Supporting Information for a detailed schematic of the thermal behavior of HE100). Therefore, it is concluded that the origin of the self-healing of FE50 is not the thermally induced flow effect of the polymeric chains.

Based on these results, the self-healing mechanism of FE50 was derived as follows. As mentioned earlier, specific interactions between the polymeric chains are the crucial driving force required to induce effective self-healing [42,43,44]. Unlike HE100, PEEK, PSU, and PES, FE50 contains electron donors and acceptors that are distributed alternately along the polymeric chain. When the surface of FE50 is scratched, the electron donors and acceptors are exposed on the surfaces of the two walls separated by the damaged area, as shown in Scheme 2. As the self-healing process begins, the electron donors and acceptors on the two walls begin to reinitiate the formation of multiple CTCs from the bottom of the scratch. As the reformation of CTCs continues, newly generated strong CTC interactions between the FE50 chains on the two walls trigger self-healing.

In contrast, because CTC interactions do not exist in HE100, the scratched area on the surface of HE100 does not self-heal, and the damaged area remains even after 12 h of self-healing, as shown in Figure 7. Furthermore, CTC interactions can significantly enhance the interchain interactions in FE50, effectively eliminating the typical trade-off of conventional self-healing materials. Thus, FE50 has superior bulk and surface mechanical properties compared with conventional EPs, as shown in Figure 5 and Figure 6.

## 4. Conclusions and Future Perspectives

In summary, self-healable PCMs based on CTC interactions were prepared in this study. The CTC-based PCMs showed good thermal stability (no significant weight loss or thermal decomposition under 350 °C) because of CTC-induced π–π stacking interactions. The CTC-based PCMs also exhibited more rapid self-healing kinetics and superior self-healing efficiency (98.1% healing efficiency within 1 min) than conventional EPs because of the strong CTC interactions between the polymeric chains. In addition, by tightly packing the polymeric chains through CTC interactions, the CTC-based PCMs exhibited significantly improved bulk (tensile strength of 106.2 MPa and the second highest Young’s modulus of 2.0 GPa) and surface (nanoindentation hardness of 386.0 MPa and nanoindentation modulus of 5.3 GPa) mechanical properties. Consequently, the CTC-based self-healing PCMs prepared in this study achieved excellent self-healing performances and mechanical strengths by overcoming the trade-off experienced by conventional self-healing materials. The hard coating materials traditionally used to protect the surfaces of flat displays cannot be used for flexible displays because of their brittle characteristics. Therefore, this study can provide a route for developing new functional PCMs to effectively protect the surfaces of next-generation displays.

## Figures and Tables

**Table 1:** Summary of compositions and thermal properties of FE100, FE75, FE50, FE25, and HE100.

Sample Designations	Dianhydride [%]		Diamine [%]		Solid Contents [%]	*T*_d_^a^ 2% [°C]	*T*_g_^b^ [°C]
	6FDA	HPMDA	ODA	EDBEA			
FE100	100	-	-	100	20	352.8	120.1
FE75	100	-	25	75	20	391.7	151.3
FE50	100	-	50	50	20	397.5	187.6
FE25	100	-	75	25	20	402.0	240.0
HE100	-	100	-	100	20	-	92.6

^a^ Thermal decomposition temperature; ^b^ glass transition temperature.

**Table 2:** Summary of mechanical and thermal properties of FE75, FE50, PEEK, PSU, and PES.

Sample Designations	Tensile Strength [MPa]	Elongation at Break [%]	Young’s Modulus [GPa]	HIT ^a^ [MPa]	EIT ^b^ [GPa]	*T*_g_^c^ [°C]
FE75	81.7 ± 6.4	4.2 ± 0.4	2.0 ± 0.1	-	-	151.3
FE50	106.2 ± 8.1	6.5 ± 0.9	2.0 ± 0.1	386.0	5.3	187.6
PEEK	90.1 ± 4.1	155.8 ± 15.1	2.3 ± 0.3	331.0	4.8	142.8
PSU	66.5 ± 0.4	24.3 ± 6.9	0.3 ± 0.1	383.3	4.9	190.0
PES	79.4 ± 0.6	27.5 ± 0.7	0.4 ± 0.2	85.9	1.1	180.0

^a^ Nanoindentation hardness; ^b^ nanoindentation modulus; ^c^ glass transition temperature.

## Supplementary Materials

The following supporting information can be downloaded at: https://www.mdpi.com/article/10.3390/polym14235181/s1. Figure S1. Schematic illustration of the CTC formation. D and A represent the electron donor and the electron acceptor, respectively. Figure S2. DSC thermogram of HE100. Figure S3. Detailed schematic illustration of the thermal behavior of HE100.
