# Supplementary material for: Highly Self-Healable Polymeric Coating Materials with Enhanced Mechanical Properties Based on the Charge Transfer Complex
Source: Polymers (Basel). 2022 Nov 28;14(23):5181. doi: 10.3390/polym14235181 (PMC9738447; doi:10.3390/polym14235181)
Supplement: Supplementary file 1 [file polymers-14-05181-s001.zip › polymers-1991021-supplementary.pdf]

Supporting Information

Highly Self-Healable Polymeric Coating Materials with Enhanced Mechanical Properties based on The Charge Transfer Complex

Chanjae Ahn [1,2,†], Pyong Hwa Hong [3,†], Juhen Lee [1], Jinsil Kim [3,4], Gyeongmin Moon [3,5], Sungkoo Lee [3], In Park [3], Haksoo Han [2,*], and Sung Woo Hong [3,6,*]

[1] Organic Materials LAB, Samsung Advanced Institute of Technology, 129 Samsung-ro, Yeongtong-gu, Suwon-si, Gyeonggi-do 16677, Republic of Korea

[2] Department of Chemical and Biomolecular Engineering, Yonsei University, 50 Yonsei-ro, Seodaemun-gu, Seoul 03722, Republic of Korea

[3] Intelligent Sustainable Materials R&D Group, Korea Institute of Industrial Technology, 89 Yangdaegiro-gil, Ipjang-myeon, Seobuk-gu, Cheonan-si, Chungcheongnam-do 31056, Republic of Korea

[4] Department of Chemical Engineering, University of Montreal, 2900 Edouard Montpetit Blvd, Montreal, Quebec H3T 1J4, Canada

[5] Current Address: MS Development Team, PI Advanced Materials, 27 Godeung 1-gil, Iwol-myeon, Jincheon-gun, Chungcheongbuk-do 27818, Republic of Korea

[6] Convergence Research Center for Solutions to Electromagnetic Interference in Future-mobility, Korea Institute of Science and Technology, Seoul 02792, Republic of Korea

* Correspondence: hshan@yonsei.ac.kr (H.H.); swhong@kitech.re.kr (S.W.H.); Tel.: +82-2-2123-2764 (H.H.); +82-41-589-8675 (S.W.H.)

† These authors contributed equally to this work.

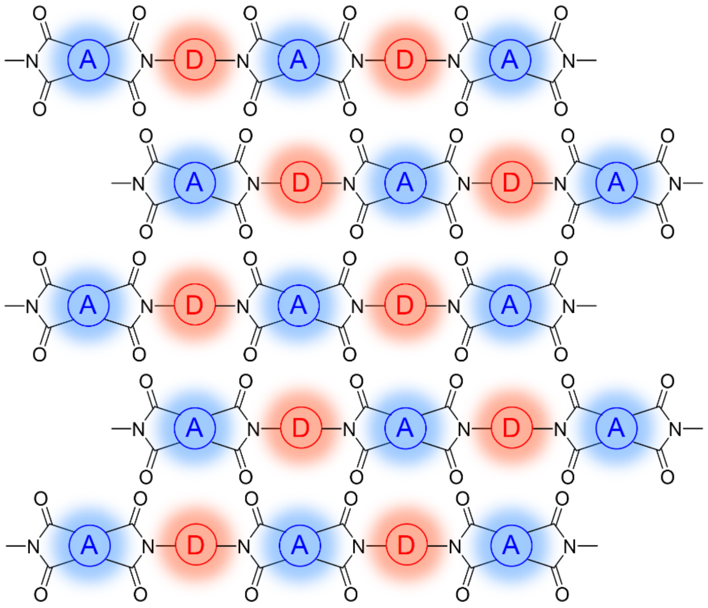

Figure S1. Schematic illustration of the CTC formation. D and A represent an electron donor and an electron acceptor, respectively.

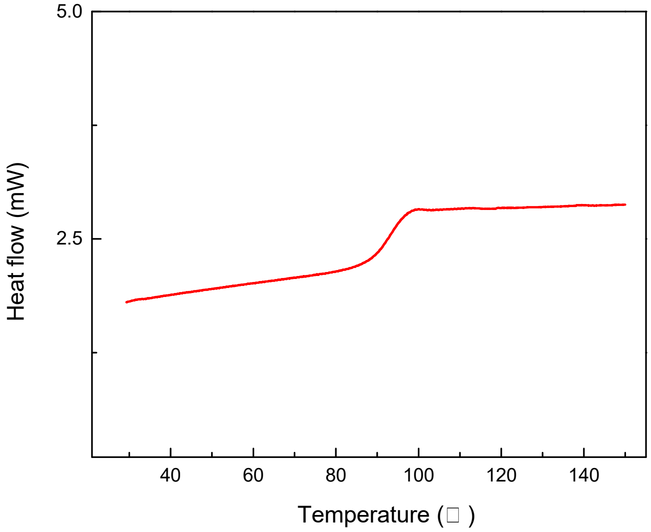

Figure S2. DSC thermogram of HE100.

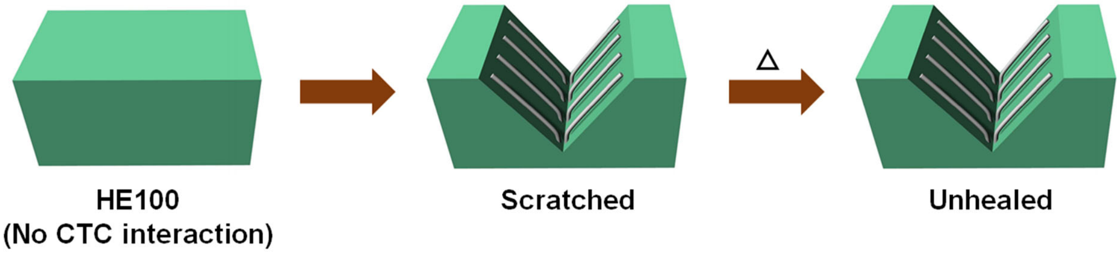

Figure S3. Detailed schematic illustration of the thermal behavior of HE100.
